# Contralateral Massive Neonatal Arterial Ischemic Stroke Secondary to Carotid Artery Dissection: A Case Report

**DOI:** 10.7759/cureus.48452

**Published:** 2023-11-07

**Authors:** Marwa M Elgendy, Josef Cortez

**Affiliations:** 1 Neonatology, University of Florida College of Medicine – Jacksonville, Jacksonville, USA

**Keywords:** shoulder dystocia, seizures, forceps, dissection, apnea, stroke, internal carotid artery, full term neonates

## Abstract

Carotid artery dissection is an infrequent cause of neonatal-perinatal stroke. Its rarity may be due to underdiagnosis or lack of awareness. We report a case of a full-term, large-for-gestational-age (LGA) male infant delivered at 39 weeks gestation. Pertinent prenatal and perinatal history include gestational diabetes, preeclampsia, and the use of forceps to assist in delivery due to shoulder dystocia. The infant presented with apnea and cyanosis while rooming which prompted admission to the neonatal intensive care unit (NICU). Initial sonographic investigation revealed an infarct, subsequently confirmed as a massive left-sided infarct by magnetic resonance imaging (MRI) of the brain. Further, computerized tomography (CT) angiography confirmed a dissection in the right common and internal carotid arteries. The child was treated with antiepileptic and antithrombotic medications. He is now undergoing regular neurodevelopmental monitoring and rehabilitation. As per our sources, this case is the first to report a contralateral significant perinatal stroke due to carotid artery dissection. It underscores the importance of recognizing subtle signs of neonatal encephalopathy that may be due to perinatal stroke, of which carotid artery dissection is an uncommon etiology. Assisted delivery techniques such as the use of forceps may be risk factors.

## Introduction

Acute perinatal stroke occurs, on average, in 1:1,100 livebirths, and is a significant neonatal neurological disorder, resulting from disruptions in cerebral blood flow due to arterial or cerebral venous thrombosis or embolization, which can occur from the 20th week of fetal life to the 28th day post-birth [1,2]. Manifestations in newborns can be immediate, as seen in unexplained encephalopathy, or may surface later in childhood. Notably, newborns presenting with signs of neonatal encephalopathy such as seizures, lethargy, hypotonia, feeding difficulties, apnea consequently leading to hypoxia and cyanosis, or distinct neurological deficits warrant an evaluation for perinatal stroke [3].

The pathophysiology underlying perinatal strokes remains incompletely understood. Their development can be attributed to a complex interplay of maternal, placental, fetal, and neonatal factors. Perinatal arterial ischemic stroke mechanisms are broadly categorized into the following: emboli from cardiac and extra-cardiac sources, thrombosis from imbalanced homeostasis, and arteriopathies. Yet, for a considerable number of acute perinatal stroke cases, identifying a definitive causative factor remains challenging [4-6].

Arteriopathies, although uncommon, are pivotal considerations in arterial ischemic strokes, especially when the root cause of an acute perinatal stroke remains elusive [7,8]. Confirmatory diagnosis typically relies on detailed neuroimaging or neuropathological examinations [3]. Unfortunately, many perinatal stroke survivors experience long-term morbidities, encompassing cerebral palsy, epilepsy, learning disabilities, cognitive and behavioral issues, and visual impairments [9].

## Case presentation

Clinical presentation and maternal history

The presented case involves a full-term male infant who was classified as large for gestational age (LGA) and was delivered vaginally at 39 weeks 2 days of gestation. The mother, a 30-year-old woman with a history of three pregnancies and deliveries (G4 P3), received adequate prenatal care. However, her pregnancy was complicated by the coexistence of gestational diabetes (controlled by an oral hypoglycemic agent and did not require insulin) and preeclampsia. Maternal serology tests and other prenatal testing were unremarkable. There was no history of chorioamnionitis or maternal history of connective tissue diseases. The delivery was complicated by meconium staining and the use of forceps due to shoulder dystocia. Despite these complications, the neonate was vigorous at birth with Apgar scores of 7 and 9 at one and five minutes, respectively. The birth weight was 4.2 kg. The newborn was subsequently left with his mother for mother-baby bonding and breastfeeding.

Respiratory distress and initial management

At three hours of life, the infant began having apneic and cyanotic spells requiring oxygen support via nasal cannula with a fraction of inspired oxygen (FiO_2_) of 0.03 to maintain oxygen saturations above 94%. A chest x-ray was conducted, revealing mild haziness but was generally unremarkable. Early-onset sepsis was ruled out after blood culture results came back as negative after 36 hours and serial complete blood count (CBC) determination was normal. The serum electrolytes profile was also normal. The patient continued to have apneic and cyanotic spells briefly requiring non-invasive positive pressure ventilation. Overt clinical seizures were not observed at this time.

Diagnostic investigation and cardiac findings

Given the persistence of the spells, further diagnostic assessments were conducted. Repeated chest x-ray did not reveal pneumothorax or other pulmonary abnormalities. Echocardiography detected a 4-mm secundum atrial septal defect (ASD), a trivial patent ductus arteriosus (PDA) with left to right shunt, no ventricular abnormalities, and normal biventricular function.

Neurological evaluation and imaging

**Figure 1:**
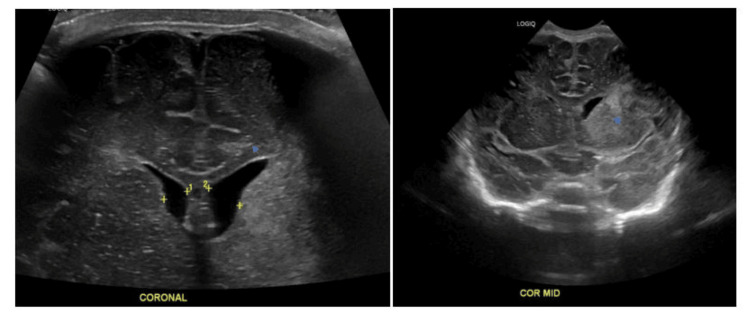
Findings of cranial ultrasound/coronal view. (Left, right) An abnormal echogenicity in the left deep gray matter and left periventricular white matter concerning ischemia/infarct. Concern for infarct/ischemia with some mild L>R midline shift as illustrated by the arrows.

**Figure 2:**
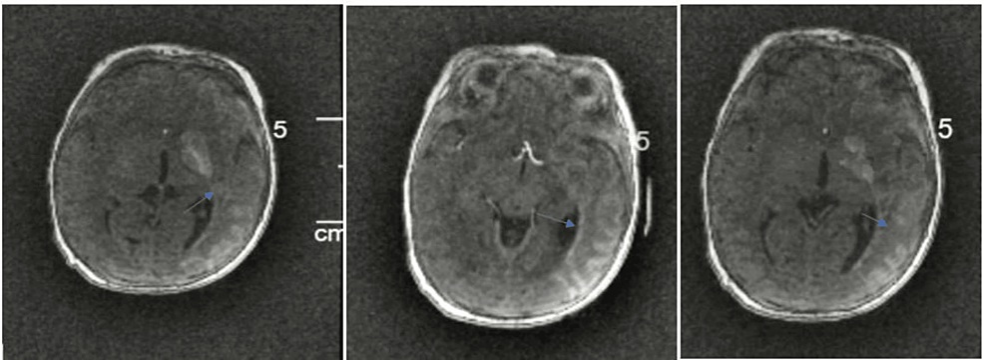
MRI and MRA findings MRI and MRA brain demonstrated a large infarct involving the entire territory of the left middle cerebral artery, involving the left frontal, temporal, and parietal lobes, insula, and basal ganglia that is being shown by the arrows. A much smaller infarct is demonstrated in the right MCA territory in the right temporal lobe and small area of the right parietal lobe. T2 hyperintense edema and swelling are demonstrated throughout the left MCA territory. No midline shifts. Ventricles are normal in size.

A head ultrasound was performed on the first day of life, indicating concerns about infarct/ischemia and a midline shift (Figure 1). This finding prompted obtaining a brain MRI which confirmed a substantial infarction encompassing the entire territory of the left middle cerebral artery. Additionally, a smaller infarct was observed on the right side. This left-sided infarct resulted in T2 hyperintense edema and swelling within the left MCA territory. Minimal hemorrhage was also identified on susceptibility-weighted images (Figure 2).

Neonatal stroke mechanism and thrombophilia evaluation

**Figure 3:**
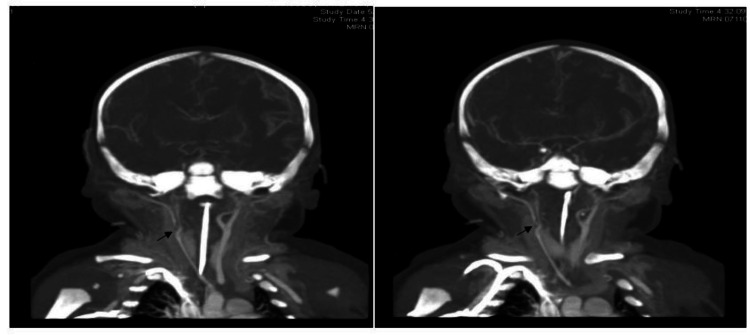
Computerized tomography (CT) angiography findings (Left, right) CT angiography demonstrated diffusely small caliber of the right common carotid and internal carotid arteries. Focal filling defect at the carotid bifurcation, thrombus or dissection that is being shown by the arrows.

The patient's neonatal stroke was likely precipitated by either a thromboembolic phenomenon originating from the placenta or by direct vessel injury during birth. Despite the pursuit of thrombophilia studies including antithrombin, protein C and protein S, factor V Leiden, and homocystinuria, no evidence of maternal or infant thrombophilia was detected. A CT angiography of the neck was conducted to assess the potential for direct carotid artery injury, given the complexity of the delivery. The results demonstrated the reduced caliber of the right common and internal carotid arteries, with a focal filling defect suggestive of thrombus or dissection (Figure 3).

Management and follow-up

The infant was eventually found to have subclinical seizures. He was started on an antiepileptic (levetiracetam) and prescribed antithrombotic therapy for a duration of six weeks. The patient is currently being followed up regularly in an outpatient neurodevelopmental and rehabilitative clinic.

## Discussion

Our study presents a remarkable case of a full-term infant delivered vaginally with forceps assistance due to complications associated with gestational diabetes and macrosomia. The infant exhibited non-specific signs of cyanosis and apnea, important as these subtle perturbations may very well be the initial signs of neonatal encephalopathy, especially in the absence of any apparent infectious, cardiac, or respiratory conditions. Through investigation including imaging studies, we identified the underlying cause to be an acute perinatal stroke of massive left-sided brain infraction associated with a traumatic dissection of the right internal and right common carotid artery. The current study is the first to report the contralateral massive perinatal stroke in neonates with traumatic carotid artery dissection.

The spectrum of acute symptomatic perinatal strokes encompasses three distinct categories: neonatal arterial ischemic stroke (NAIS), neonatal cerebral sinovenous thrombosis, and neonatal hemorrhagic stroke [1,2]. In our case, the manifestation aligns predominantly with NAIS. Typically, acute symptomatic perinatal strokes present with focal seizures occurring shortly after birth [10]. In the context of our study, the infant's eventual diagnosis of subclinical seizures aligns with this characteristic presentation. This initial symptomatology often serves as a key indicator prompting further investigation. In our presented case, the neonate displayed distressing signs, including episodic apnea and cyanosis, which in retrospect could be seizure manifestation. Notably, these symptoms persisted despite thorough investigations ruling out infectious, cardiac or respiratory factors as the underlying cause. Given the presence of multiple apneas and the absence of apparent infectious, respiratory or cardiac causes, it becomes crucial to rule out seizures as a potential contributing factor. This requires vigilant diagnostic efforts. Noninvasive brain imaging studies, notably head ultrasonography (US) and magnetic resonance imaging (MRI) of the brain are pivotal in identifying abnormal brain tissues in neonates. Among these, head US serves as an initial and reliable bedside screening tool for detecting potential brain abnormalities. Its accessibility and effectiveness make it a valuable diagnostic modality [11-13].

The causes of neonatal stroke are diverse, predominantly stemming from maternal, placental, and neonatal factors. It is crucial to explore and rule out carotid artery dissection in infants diagnosed with neonatal stroke, especially when maternal and placental factors are absent, and if assistive delivery techniques such as vacuum or forceps extraction are used.

The left and right common carotid arteries bifurcate between the third and fourth cervical vertebrae, forming the internal and external carotid arteries. Notably, the internal carotid artery facilitates the anterior circulation of the brain, including the anterior and middle cerebral arteries [14].

Carotid artery dissection, while a rare occurrence, has emerged as an intriguing etiological factor in NAIS. This condition involves the separation of the layers of the carotid artery, a phenomenon that can arise either spontaneously or due to trauma [15]. This separation has the potential to compromise blood flow to specific regions of the brain, thereby setting the stage for the development of a stroke [15]. It is important to note that carotid artery dissections can take place either within the cranial cavity (intracranially) or outside it (extracranially), and in some cases, can even lead to the occurrence of subarachnoid hemorrhages or brain ischemia [16]. The underlying causes of carotid artery dissection vary, with trauma being the most common trigger. While trauma is a predominant cause, instances of spontaneous carotid artery dissection have been reported, although they are less frequent [17]. In our case, the neonate experienced shoulder dystocia during delivery, and the use of forceps may have further compounded the delivery's complexity. These factors may potentially lead to direct trauma to the carotid vessels. Additionally, other mechanisms such as stretch injuries to arteries supplying the brain may have contributed. The presence of shoulder dystocia during delivery may cause traction on the carotid vessels, particularly during the process of head and shoulder delivery [15]. Furthermore, vasospasm due to compression, especially in the context of forceps-assisted delivery, could also play a role in carotid artery dissection [18].

To date, the available case reports detailing instances of carotid artery dissection in neonates are limited to nine patients [18]. Interestingly, all nine of these cases share a common characteristic: neither the neonates nor their mothers exhibited any fetal problems or maternal thrombophilia. This observation suggests that the occurrence of carotid artery dissection in neonates may not always be linked to preexisting medical conditions. Dissection of the internal carotid artery has been observed to occasionally lead to ipsilateral brain infarction and stroke, as documented in all adult studies and the nine published neonatal studies [19]. However, our research presents a novel observation of contralateral massive left-sided brain infarction.

The current study highlights a rare case featuring a contralateral massive left-sided brain infarction, with a significant infarct spanning the entirety of the left middle cerebral artery's territory. This encompasses the left frontal, temporal, and parietal lobes, insula, and basal ganglia. Additionally, a minor infarct was observed in the right MCA territory, affecting the right temporal lobe and a segment of the right parietal lobe. These anomalies can be attributed to the traumatic dissection of the right common and right internal carotid artery.

In such clinical scenarios, ischemia typically ensues due to an impairment in the collateral blood flow through the circle of Willis. This crucial vascular structure acts as a communicative pathway, ensuring balanced blood flow between both cerebral hemispheres and facilitating anastomotic circulation [14]. If a dissection occurs in the internal carotid artery, it may lead to reduced blood flow or a complete blockage. In an optimal situation, the other arteries within the circle of Willis would compensate for this loss. However, not everyone has a complete and robust circle of Willis. Some people have congenital variations or underdeveloped arteries within this circle. If the collateral pathways within the circle of Willis are inadequate or compromised, it may not sufficiently compensate for the loss of blood flow. The exact mechanism of contralateral stroke would depend on individual anatomical variations, the extent of the dissection, and other hemodynamic factors [14,18]. Another mechanism involves hemodynamic changes, the reduced pressure on the side of the dissection can alter blood flow dynamics within the circle of Willis, potentially diverting blood away from the contralateral side and leading to ischemia [14].

CT angiography is the preferred imaging method for diagnosing carotid artery dissection. However, in studies focused on neonatal stroke, angiographic techniques, especially at the cervical level, are not commonly included in protocols, even though their importance has been previously emphasized [19]. Acquiring these images in neonates presents numerous challenges, particularly if the neonates are unstable or critically ill. Furthermore, interpreting angiographic findings in neonates requires the expertise of specialized radiology teams.

Carotid artery dissections in cases of neonatal stroke are likely underdiagnosed. Suspicion of carotid artery dissection as an underlying cause of neonatal stroke arises when a patient exhibits seizures and abnormal neurological signs shortly after birth. This suspicion increases if the patient displays extensive ischemic damage on MRI scans and possesses risk factors like traumatic or instrumental delivery.

The management approach for NAIS primarily involves supportive care. This includes controlling seizures, optimizing oxygenation, maintaining adequate blood volume, and addressing fluid balance. While antiplatelet medications like aspirin and anticoagulation with low-molecular-weight heparin (LMWH) or unfractionated heparin (UFH) are rarely prescribed due to the low risk of recurrent stroke following NAIS, in some cases, LMWH might be initiated for a total duration of six weeks if extensive infarction is observed in MRI results [20].

Our patient was treated with anticoagulant for total of six weeks. The medical management of acute perinatal stroke caused by carotid dissection is still a topic of debate. The routine use of LMWH and antiplatelet agents in neonatal stroke cases is not recommended unless in cases showing clinical deterioration or evidence of thrombus extension on serial imaging [20].

Anticoagulant treatment might be considered in neonates at risk of stroke recurrence due to severe thrombophilia or cerebral embolism resulting from cardiac issues or in neonates with cerebral venous sinus thrombosis (CSVT) [20]. However, it is generally not advised for neonates experiencing their first ischemic infarction.

This case study has the novel findings of contralateral stroke occurrence as a result of internal carotid artery dissection and underscores the intricate interplay between maternal complications, delivery complexities, neonatal distress, and the subsequent diagnosis and management of neonatal stroke, with emphasis on dissection of carotid arteries as a potential mechanism. Further research and clinical experience will refine our understanding of neonatal stroke mechanisms and guide optimal management strategies.

## Conclusions

The dissection of carotid arteries may be a significant contributor to the etiopathogenesis of NAISs. Such dissections can lead to not only ipsilateral perinatal strokes but also contralateral ones. When neonatal strokes occur, especially in the absence of maternal or placental causes and following traumatic births, it is crucial to consider carotid artery dissection as a potential cause. Anticoagulant medications have shown promise as an effective treatment for these neonates, and they have been associated with favorable neurodevelopmental outcomes. As such, it is essential to establish neonatal guidelines that emphasize the importance of prompt detection and management of carotid artery dissection in newborns.
